# Directly Probing Light Absorption Enhancement of Single Hierarchical Structures with Engineered Surface Roughness

**DOI:** 10.1038/s41598-018-29652-8

**Published:** 2018-08-16

**Authors:** Jingwei Wang, Run Shi, Weijun Wang, Nianduo Cai, Pengcheng Chen, Dejun Kong, Abbas Amini, Chun Cheng

**Affiliations:** 1 Department of Materials Science and Engineering, Southern University of Science and Technology, Shenzhen, 518055 China; 2 Center for Infrastructure Engineering, Western Sydney University, Kingswood, New South Wales 2751 Australia

## Abstract

Hierarchical nanostructures are ideal architectures to harvest solar energy. The understanding of light absorption in single hierarchical structures is emergently important and greatly helpful in enhancing multiscale optical phenomena and light management. However, due to the geometrical complexity of hierarchical architectures, theoretical and experimental studies of light absorption have faced significant challenges. Here, we directly quantify light absorption in single hierarchical structures for the first time by utilizing VO_2_-based near field powermeter. It is found that light trapping is significantly enhanced in rough microwires when the roughness amplitude is comparable to the incident light wavelength. The roughness enhanced light absorption is verified as a general phenomenon on both VO_2_ and Si hierarchical structures. Therefore, our work not only provides a simple and quantitative method of measuring light absorption upon single geometrically complex structures in micro/nanoscale, but also contributes a general rule to rationally design of hierarchical structures for enhanced performance in photoelectric and photochemical applications.

## Introduction

Conversion of sunlight to electrical and chemical energy is a promising and proven strategy for large-scale production of energy from a renewable source. To effectively harness solar energy, a photovoltaic cell or photoelectrochemical electrode must absorb most of the solar spectrum and collect the photon-generated carriers with minimal losses to recombination. For planar devices, this task can be difficult because the required thickness of material for adequate absorption of light is often greater than the distance over which photon-generated charges can be efficiently collected. Semiconductor micro/nanostructures offer new approaches to meet these requirements of light absorption and charge collection^1–3^. Specifically, hierarchical architectures that are constructed by multiscale micro/nanostructures have attracted intensive attentions since they are ideal candidates for high-performance solar energy harvesting devices^4–8^. These structures possess specific configurations of microscale backbones and one-dimensional (1D) nanostructure arrays surrounded the backbones. The direct pathway along 1D crystalline nanostructures diminishes the possibility of charge recombination and offers a relatively large aspect ratio for rapid electron-hole separation and charge transport, as well as electrochemical reactions^9–11^. Most importantly, high-density treelike branched nanowire arrays of hierarchical structures provide long optical paths for efficient light trapping and thus promote the utilization of light significantly^12–16^. Plenty of efforts have been devoted to the synthesis and applications of hierarchical structures, while how their structural parameters, such as the size of multiscale nanostructures, affect light absorption is yet to be systematically studied. Thus, quantification of light absorption upon single hierarchical structures has to be settled urgently.

Light absorption and propagation at the sub-micrometer scale are the key points for many critical technologies and applications. Light absorption of 1D nanostructure arrays is a well understood phenomenon experimentally, analytically (Maxwell equation), and numerically (finite element method). However, the light absorption of single sub-micron solids is still hard to be quantitatively determined by experiments^16–18^. This is largely due to difficulties in accurate and direct characterization of energy flow and temperature distribution at this scale. In case of geometrically complicated nanostructures, *i*.*e*., hierarchical structures, simulational or experimental evaluations of light absorption become even more difficult.

Recently, we have developed optically readable near-field powermeters (NFP) based on the metal–insulator transition (MIT) in single crystal vanadium dioxide (VO_2_) micro/nanobeams which enables direct quantification of light absorption of any single micro/nanowires^19,20^. Here, we present the quantitative study of light absorption of single hierarchical structures by applying this unique NFP technique. The surface of VO_2_ and Si microwires were engineered by a top-down approach with focus ion beam (FIB) technique to form hierarchical structures with controllable trench sizes. Long VO_2_ wires were suspended from a substrate as NFPs. The hierarchical wires bonded to the NFPs were locally heated using a focused laser which stimulates the phase transition. The resultant domain structures can be optically imaged. By using the heat transport theory, we determined the light absorption as a function of trench depth and spacing. It was found that light trapping was significantly enhanced for hierarchical structures (rough micro/nanowires) when the amplitude of surface roughness was comparable to the light wavelength. From the results, an enhanced light absorption was obtained from the single hierarchical structures prepared by surface roughness engineering. It is found that the enhanced light absorption could be obtained by surface roughness engineering on both VO_2_ and Si material systems which thus provides a universal strategy for modern hierarchical structure design.

## Methods

For the optical absorbance measurements of single VO_2_ and Si microwires with patterned trenches, experiments were carried out in a chamber with a quartz window (See the Supplementary Files for the details of sample and device preparation)^19–21^. The vacuum chamber was pumped down to <10^−3^ torr by a mechanical pump. Optical images of devices were recorded using an optical microscope equipped with a CCD camera. The laser heating of cantilevered devices was carried out using the microscope with continuous-wave Ar ion laser at the wavelength 488 nm (Horiba, HR Evolution). The stage was movable in the x-y plane with respect to the laser spot, and the laser power was tuned by an assembly of beam splitter and neutral filters. The size of the focused laser spot was less than 2 μm. The safe maximum laser power was determined when the visible permanent damage was observed on the surface of the VO_2_ or Si nanowire with the focused laser, then, much lower laser intensities (<100 mW) were used in subsequent experiments. To eliminate the misalignment in each measurement, the laser focal depth and position with respect to the nanowire were carefully adjusted until the maximum M domain length was reached at the VO_2_ NFP. The laser polarization direction was kept parallel to the axis direction of the studied nanowire to eliminate the influence of polarization. The total incident laser power was measured using a commercial powermeter (Thorlabs, S120C). In this study, the MIT temperature Tc is 68 °C, the room temperature Ts is 25 °C and the cross section of applied VO_2_ wire A is 16 µm^2^, respectively.

## Results and Discussion

**Figure 1:**
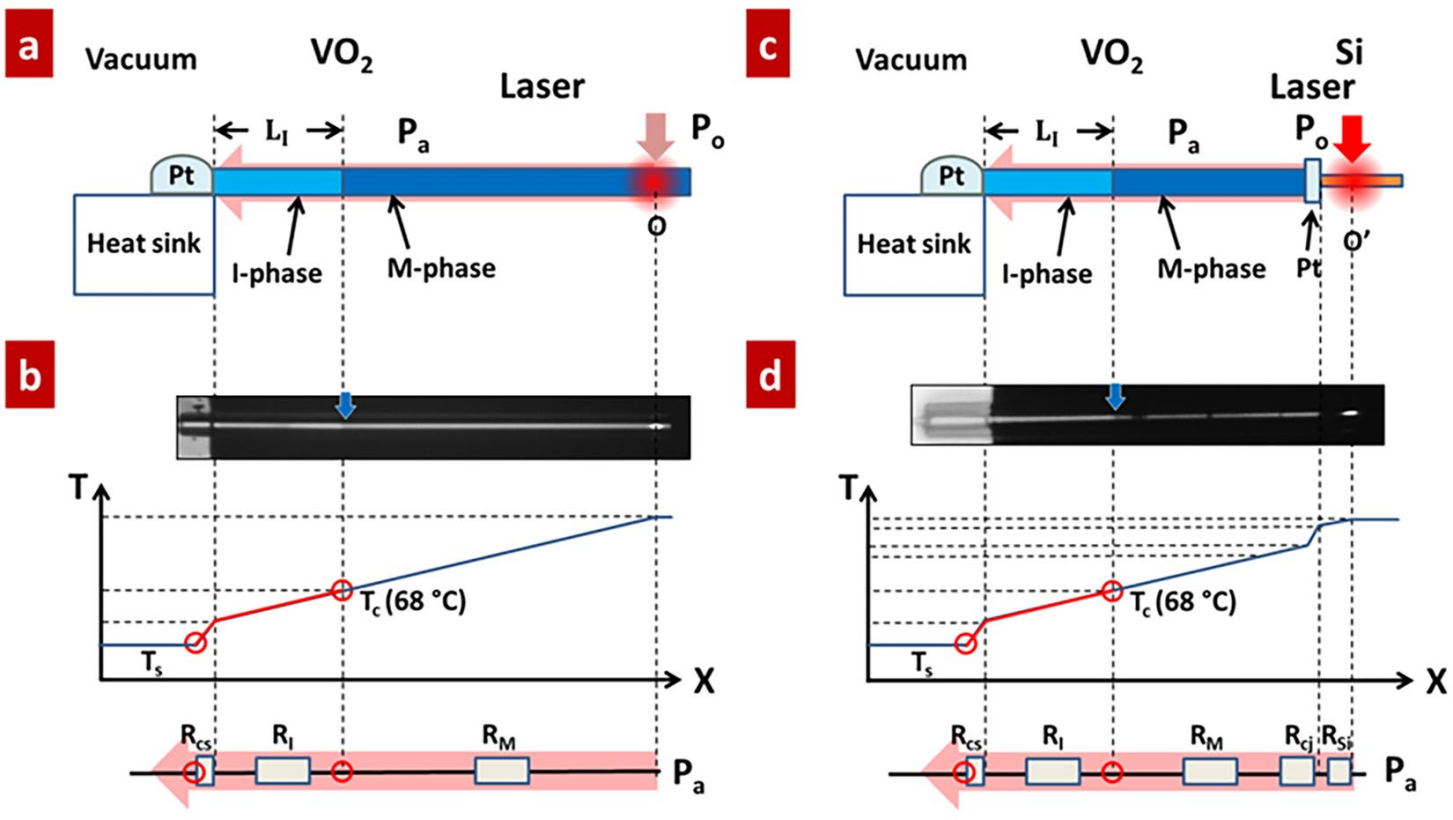
Working principle of the near-field powermeter (NFP). (**a**) A focused laser beam thermally activates a metal domain in a cantilevered VO_2_ micro/nanowire. The optically read M/I domain wall indicates the position at the MIT temperature, *T*_C_. (**b**) Schematic of temperature profile along the VO_2_ microwire. Between (**a** and **b**) is an image of a heated VO_2_ microwire. (**c**) Quantifying optical absorbance of a single Si nanowire. The NFP is bonded to a Si nanowire, allowing quantification of optical absorption of the single Si nanowire. (**d**) Schematic of temperature profile along the Si nanowire-VO_2_ NFP system. Between (**c** and **d**) is an optical image of a heated Si nanowire. Heat was unidirectionally transferred to the substrate through the VO_2_ NFP and triggered the MIT, leaving a distinguishable M/I domain wall. The red arrow indicates the positions that the laser beam focuses on and the blue arrows mark the positions of M/I domain wall.

Figure 1a,b shows the configuration of a VO_2_ microwire based NFP for the quantitative measurement of light absorption. A VO_2_ wire was cantilevered at the edge of a heat sink (silicon substrate); its root was deposited by FIB with Pt to ensure a good thermal contact. A continuous 488 nm-wavelength laser is focused at a targeted position along the microwire and provides local heating. The whole system (Fig. 1a) was placed in vacuum (<~1 × 10^−3^ torr), so that the heat dissipation to air was negligible compared with that to the big heat sink. After increasing the laser intensity beyond a threshold, obvious temperature gradient was observed across the region between the heat sink and heating positions (Fig. 1b). For a single VO_2_ wire with temperature above *T*_*C*_, the insulator phase changed to metal phase leaving an M/I domain wall between the laser spot and the root. These two phases across the M/I domain wall are distinguished differently from light reflection. Hence, the single VO_2_ wire works as a micro/nanoscale powermeter where the M/I domain wall can be optically read out to specify the positions with local temperature of *T*_*C*_.

For measuring the light absorption of a VO_2_ wire with the cross section area (A), the laser beam is focused at the top surface of the tip position of the VO_2_ wire (point O, Fig. 1a). Considering the 1D steady thermal conduction from the M/I domain wall to the heat sink (the red line section), the I-domain length *L*_*I*_ can be expressed as follows according to Fourier’s law^19^:

1 $${L}_{I}=\frac{{\rm{\Delta }}T\cdot {\kappa }_{V{O}_{2}}\cdot A}{{P}_{a}}-{R}_{cs}\cdot {\kappa }_{V{O}_{2}}\cdot A$$

Here, Δ*T* = 46 °C is the temperature difference between the domain wall (*T*_*C*_) and substrate (*T*_*S*_); ${\kappa }_{V{O}_{2}}$ is the thermal conductivity of VO_2_ (6.5 W/mK for both the M and I phases)^22^; the optical absorbance of the VO_2_ microwire is defined as *α* ≡ *P*_*a*_/(*βP*_0_), where *P*_*a*_ is the absorbed optical power flow and *P*_0_ is the total incident laser power which is measured by a commercial powermeter. *R*_*cs*_ is the contact thermal resistance at the Pt bond of heat sink. The geometrical factor *β* is the percentage of incident laser power physically striking the microwire, which is given by integrating the incident Gaussian laser beam energy over the area of microwire exposed to the laser. For the laser beam with diameter less than that of the targeted microwire, β = 1. From Equation 1, *L*_*I*_ is linear with 1/*P*_0_; the interception of this dependence with the *L*_*I*_ axis gives the thermal contact resistance *R*_*C*_, and the slope gives the absorbance *α*. Thus, by varying *P*_0_ and recording the corresponding values of *L*_*I*_, *R*_*cs*_ and *α* can be achieved by plotting *L*_*I*_ − 1/*P*_0_ line. For measuring the light absorption of a single nanowire which is bonded to the VO_2_ NFP (Si wire is taken as an example, Fig. 1c), as the light is injected on the laser spot of the wire (O′), the absorbed energy entirely flows unidirectionally toward the substrate. For this, the 1D steady thermal conduction from the M/I domain wall to the heat sink must be characterized (the red line section, Fig. 1d). The Si thermal conductivity (*κ*_*Si*_) and Si/VO_2_ junction’s thermal resistance (*R*_*CJ*_) are not required for the calculation. As a result, the light absorption *α* of the Si nanowire can be obtained using the same procedure shown in Equation 1 and Fig. 1. These measurements have been reported in our recent works^19^.

**Figure 2:**
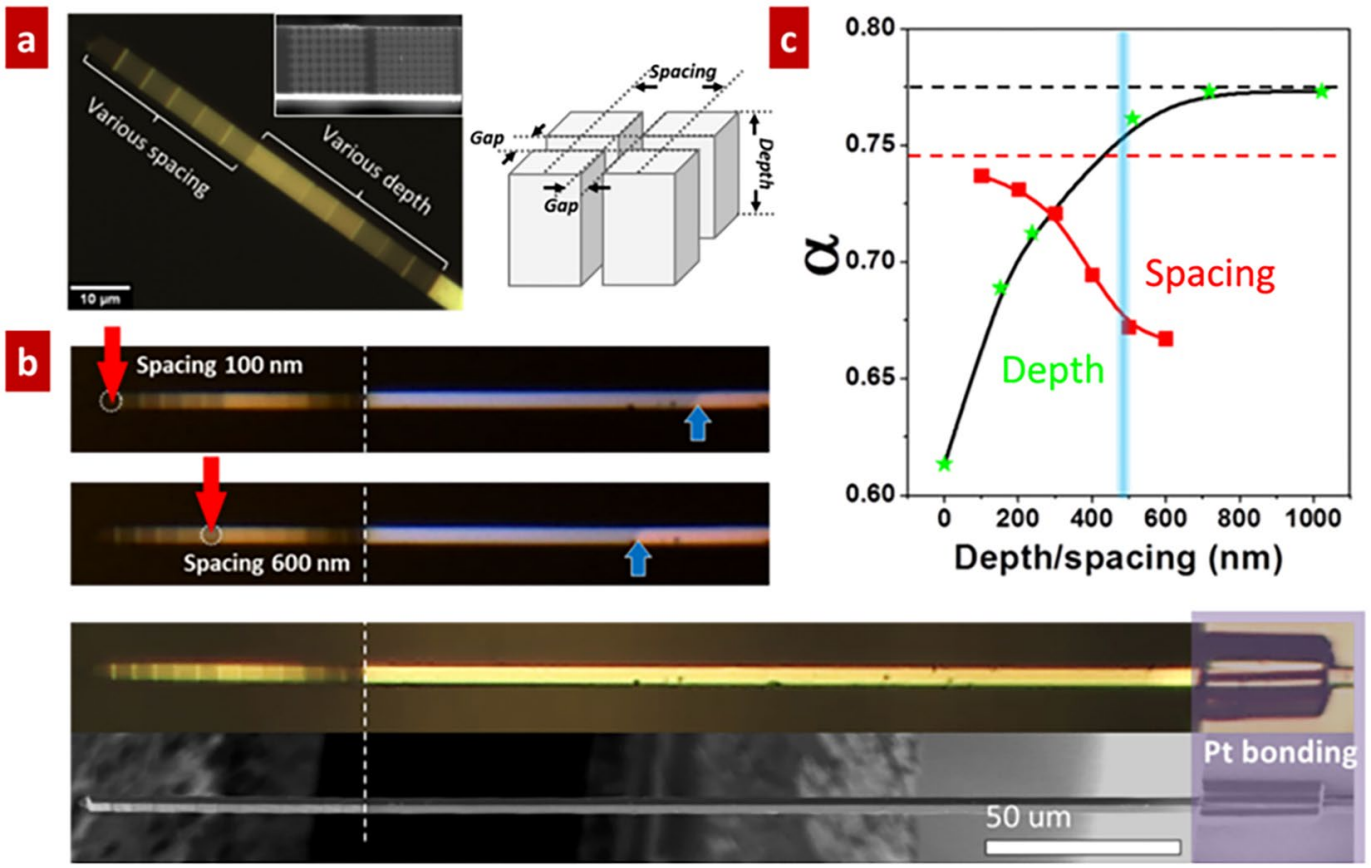
Effect of surface roughness on the optical absorption of a single VO_2_ microwire. (**a**) Optical image of a VO_2_ microwire with engineered surface roughness. The wire surface is carved with trenches of different depths and spacing using FIB. The roughened parts show a clear darker contrast than the smooth part in optical reflection, indicating effectively enhanced light absorption. The inset in the left panel is a magnified SEM image of FIB carved section. The right panel shows the configuration of the trench arrays. (**b**) The M/I wall moves towards the root of a VO_2_ microwire as the trench spacing increases (top to bottom). The images of the VO_2_ microwire bonded to the edge of Si substrate in the bottom panel are recorded by an optical microscopy and SEM (**c**) Optical absorption of a VO_2_ microwire with the width of 4.13 μm as a function of trench spacing (depth fixed at 500 nm) or trench depth (spacing fixed at 250 nm). The incident laser wavelength is 488 nm as indicated with the blue line. Solid lines and dashed lines are guidance for the eye.

After demonstrating the work principle of quantifying the optical absorbance of single micro/nanowires using VO_2_ NFPs, we study how the surface roughness affects the optical absorption of single wire with hierarchical structure. Here, VO_2_ hierarchical structures were prepared by a top-down approach using FIB. In this study, to avoid the effect of Ga ion contamination, the operation voltage was set as 10 kV. (see Supplementary Files) The tip surface of VO_2_ microwires were carved by FIB with trenches of desired depth and spacing (Fig. 2). The top two panels of Fig. 2a show the optical and scanning electron microscopy (SEM) images of a VO_2_ microwire with such engineered surface roughness. We prepare two series of trench patterns of the VO_2_ microwire: trench depths of 152, 238, 510, 718, and 1023 nm with fixed spacing of 250 nm and gap width of 40 nm (Top left Fig. 2a); and trench spacing of 100, 200, 300, 400, 500 and 600 nm with fixed depth of 400 nm and gap width of 40 nm (Bottom right Fig. 2a). Optically, the surface colour gradually darkened while decreasing the trench spacing and increasing the trench depth; these phenomena indicate that the light absorption depends strongly on the surface roughness. The VO_2_ microwire acts as the NFP and the hierarchical structure with engineered roughness surface (Fig. 2b). The bottom two panels represent the SEM and optical images of the NFP/studied system in which the wire surface is clean with similar contrast. The top two panels show the optical images of the NFP/studied system where the laser with same power are focused on two surface areas with the trench spacing of 100 and 600 nm, and fixed depth of 400 nm. Significantly distinguishable M/I domain walls are optically observed as marked by blue arrows in Fig. 2b. The M/I domain wall of those heating positions with trench spacing of 100 nm moves towards the heat sink compared to that with trench spacing of 600 nm. This fact confirms that smaller trench spacing can trap more light with similar conditions. Figure 2b represents the parameter *α* of the patterned VO_2_ wire as a function of the trench depth and spacing. This parameter strongly depends on the surface roughness while the dependency on trench parameters is yet to be confirmed. The optical absorption increases slowly with the decreasing of trench spacing firstly while it increases fast after the trench spacing is lowered than the incident light wavelength. The optical absorption gradually saturates at the value of ~0.745. The optical absorption continually increases with the trench depth. After the trench depth is larger than the wavelength of incident light, the optical absorption reaches the saturated value of about 0.775. From the above results, it is concluded that an enhanced optical absorption is attained upon the engineering surface of microwires, which is strongly correlated with the light wavelength.

**Figure 3:**
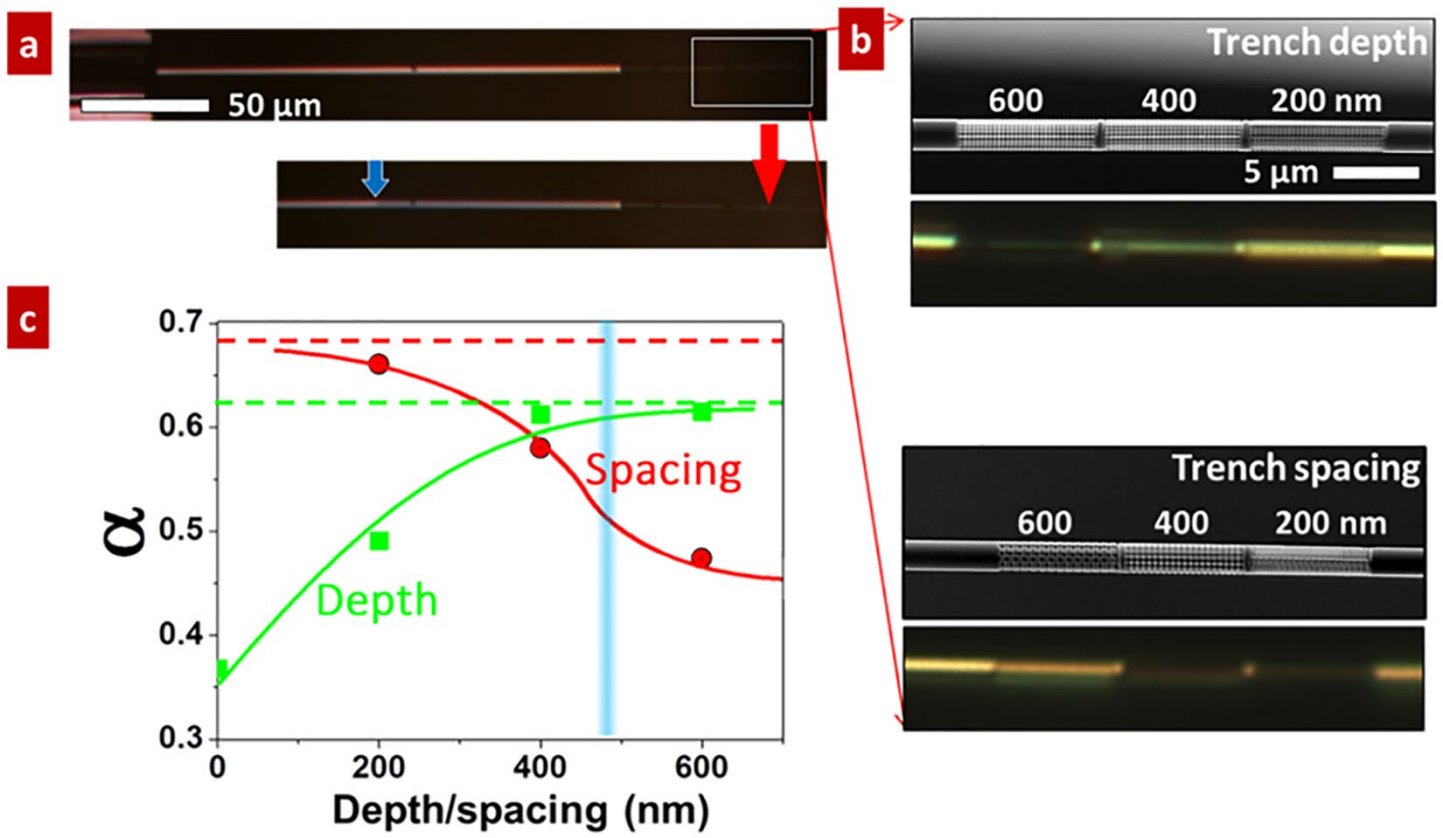
Effect of surface roughness on the optical absorption of a single Si microwire. (**a**) A surface roughness modified Si microwire bonded to a VO_2_ NFP. The left panel shows the optical images of the Si-VO_2_ system with laser off and on. A M/I domain wall is triggered when the laser is on and heat unidirectionally transfers from Si microwire to the VO_2_ NFP. (**b**) The enlarged SEM image and optical image of the patterned sections of the Si microwire. The Si microwire surface is carved with the trenches of different depths and spacing using FIB. The roughened parts show a clear darker contrast compared with the smooth part in the optical reflection, indicating effectively enhanced light absorption. (**c**) Optical absorption for a Si wire with the radius of 2.1 μm as a function of trench spacing (red circles, depth fixed at 400 nm) or trench depth (green squares, spacing fixed at 250 nm). The incident laser wavelength is 488 nm as indicated with the blue line. Solid lines and dashed lines are guidance for the eye.

To demonstrate the ability of VO_2_ NFPs for directly probing light absorption of any single micro/nanoscale complex structures, a single Si microwire is bonded onto a VO_2_ NFP (Fig. 3a). This is also to verify the universality of the conclusion that light trapping is significantly enhanced in single rough micro/nanowires when the amplitude of roughness is comparable to the light wavelength. The Si wire surface is carved by FIB with trenches and desired depth and spacing. In the bottom panel of Fig. 3a, a laser beam is focused on the Si microwire to activate the MIT of NFP; this resulted in a clearly resolved M/I domain wall. Figure 3b shows the SEM and optical images of the surface engineered sections of the Si microwire. We prepare a series of trench patterns for the Si microwire: trench depths 200, 400, and 600 nm, and fixed spacing 250 nm and gap width 55 nm; and trench spacing 200, 400, and 600 nm, and fixed depth 400 nm and gap width 55 nm. From the optical images, when the trench depth increases and trench spacing decreases, the carved zone obtains a gradual darker contrast. The measured *α* as a function of the trench depth and spacing is presented in Fig. 3c. For the pristine surface of Si microwire, *α* is 0.368. This value is close to those measured on single Si nanowires by the same approach here^18^. Deeper and narrower trenches promote *α* up to saturated values of 0.62 and 0.68, respectively. Therefore, similar to VO_2_, the *α* values of the patterned Si wire are also strongly dependent on the surface roughness and follows the same trend; higher *α* for rougher surfaces. This can be explained by the effect of enhanced light trapping in rougher wires when the amplitude of roughness is comparable to the light wavelength^23–27^. Also, the range of enhanced *α* for Si hierarchical structures correlates with the incident light wavelength.

Understanding the light absorption in single micro/nanostructures is essential for the optimization of device performance through architectural management. Up to now, the evaluation of light absorption of single micro/nanowires relies either on indirect measurement or theoretical models, which can only provide qualitative values^28–35^. Experimental methods for probing light absorption such as monitoring the photocurrent generated within single wire devices are time-consuming and require complicated microelectrodes fabrication process^36–41^. In addition, we have shown that the real nanowire absorption deviates significantly from the theoretical prediction calling for caution in using these equations or simulation results. Hierarchical structures are ideal architectures suitable for high-performance devices for solar energy harvesting and conversion^11^. Because of the geometrical complexity of hierarchical architectures, simulation or experimental evaluations of light absorption are difficult. In this study, the VO_2_-based NFP is proposed to well identify the optical absorption of single geometrically complicated micro/nanostructures by a contactless and simple optical measurement procedure, just recording the M/I domain wall position using camera. Also, as-obtained relationship between optical absorption and surface roughness of single micro/nanowires has been verified by two samples, VO_2_ and Si. From the presented results, a hierarchical structure can provide a great enhancement in optical absorption when the length and spacing of nanostructures reach certain thresholds — the incident light wavelengths. However, beyond these thresholds, longer lengths or narrower spacing does not efficiently affect the optical absorption as it quickly approaches to a certain value. Long and high dense substructures may inversely impact the performance of hierarchical structures owing to increasing recombination rate of photon-generated electron-hole pairs^42,43^. This results from the larger transport path of carriers to the backbone and more recombination centres from larger specific surface area. This study sheds the light on the rational design of sub-units of hierarchical structures based on the wavelength range of the incident light to acquire higher performance for solar energy harvesting.

## Conclusions

In this study, VO_2_-based NFPs were applied to quantitatively probe the light absorption of single VO_2_ and Si hierarchical structures with engineered surface roughness. Light trapping is significantly enhanced when the amplitude of surface roughness is comparable to the light wavelength. Considering the difficulty in theoretically evaluating optical absorption of single hierarchical structures and the discrepancy between experimental data and simulated results, we demonstrate the direct and quantitative measurement of optical absorption and thus provide a simple and advanced tool for the studies of light management upon single small and complex structures. Our results reveal a rational design rule of micro/nanostructures for enhanced light harvesting performance.

## Electronic supplementary material

Supporting Information
