# Supplementary material for: Directly Probing Light Absorption Enhancement of Single Hierarchical Structures with Engineered Surface Roughness
Source: Sci Rep. 2018 Aug 16;8:12283. doi: 10.1038/s41598-018-29652-8 (PMC6095879; doi:10.1038/s41598-018-29652-8)
Supplement: Supplementary file 1 — Supporting Information [file 41598_2018_29652_MOESM1_ESM.pdf]

Supplementary Information

Directly Probing Light Absorption Enhancement of Single Hierarchical Structures with Engineered Surface Roughness

Jingwei Wang[#,1], *Run Shi*[#,1], *Weijun Wang*[1], *Nianduo Cai*[1], *Pengcheng Chen*[1], *Dejun Kong*[1], *Abbas Amini*[2] *and Chun Cheng*[*,1]

1. Department of Materials Science and Engineering, Southern University of Science and Technology, Shenzhen, 518055, China

2. Center for Infrastructure Engineering, Western Sydney University, Kingswood, New South Wales 2751, Australia

* Corresponding Author: E-mail: chengc@sustc.edu.cn

[#] These authors contributed equally to the work.

1. Sample and device preparation

Vanadium dioxde and Si micro/nanowires synthesis: [1] V_2O_5 powder was poured in a quartz boat in the centre of a horizontal tube furnace. The reaction product was collected on substrates downstream from the source quartz boat. The growth was carried out under the following conditions: evaporation temperature ~880°C, Ar carrier gas flow rate ~6.8 sccm, pressure ~5 Torr, and evaporation time ~2 hours or more. Freestanding, single-crystal VO_2 micro/nanowires were collected from the rough (unpolished) surface of quartz substrates. Si nanowires were fabricated on a Si wafer with gold nanoparticles as the catalysts. The Si vapor source was silane gas (purity > 99.99% and flow rate 15 sccm), mixed with hydrogen (purity > 99.99% and flow rate of 100 sccm), and fed into the reaction quartz tube (under the pressure of ~50 torr) placed in a furnace. High-quality Si nanowires grew at the temperature of ~520°C.

Devices fabrication: Free standing VO_2 microwires were cantilevered to the edge of Si substrate. The Si micro/nanowires, to be measured, were transferred and coupled with the VO_2 microwires by micro probes. To reduce contact thermal resistance, Pt pads were deposited at the roots and junctions of wires using Ga^+ beam (30kV) induced deposition inside a FEI-Quanta 3D Dual Beam focused ion beam (FIB). The coupled VO_2-Si devices were loaded into a vacuum chamber for further investigation. For the preparation of Si nanowires with engineered surface roughness, the smooth surface of Si nanowire was carved using Ga^+ beam (line mode with 10pA beam

current, 10kV) in the FIB. [2]

2. Effects of Ga ion implantation on the optical absorbance

We note that the possible damage of wire surface by Ga ions irradiation may also contribute to the stronger optical absorption. So, we investigated the effect of Ga ion implantation on light absorption through various operation voltages, etching depths and time of FIB during a carving procedure. We prepared two series of Ga ion treated areas on a VO_2 microwire: the tip surface is serially milled 200 nm away using operation voltage of 5, 10, 15, and 30 kV, respectively, the sections ②-⑤ in FIG.S1a; and, the surface tip is serially milled 200 nm away 0, 10, 100, respectively, the sections ①-③ in FIG.S1b with the same FIB milling conditions (10 pA and 30 kV). FIG.S1a shows the optical images of VO_2 wire obtained from the laser beam focused at the tip surface. The blue arrow and dashed lines mark the location of the M/I domain walls triggered by laser heating. The red arrow indicates the focused position of laser. The location of the M/I domain walls did not change until the laser beam focused on the sections with the operation voltage for FIB milling larger than 10 kV. This fact indicates that Ga ion implantation with the operation voltage less than 10 kV has less impact on light absorption. When the operation voltage is beyond the threshold (10 kV), the M/I domain wall moves slightly towards the microbeam root as (FIG.S1a); this indicates the light absorption pile-up with the operation voltage. However, such enhancement effect is rapidly saturated after the 10 nm milling-depth at a fixed operation voltage of 30 kV (FIG.S1b); that is, more milling (thus, heavier

Ga dose) has no further effect on the optical absorption of microbeam. For this VO_2 wire, α increases by 0.15 at the milling zones ①-③, compared to that of the pristine surface. In this study, to avoid the effect of Ga ion contamination, the operation voltage was set as 10 kV.

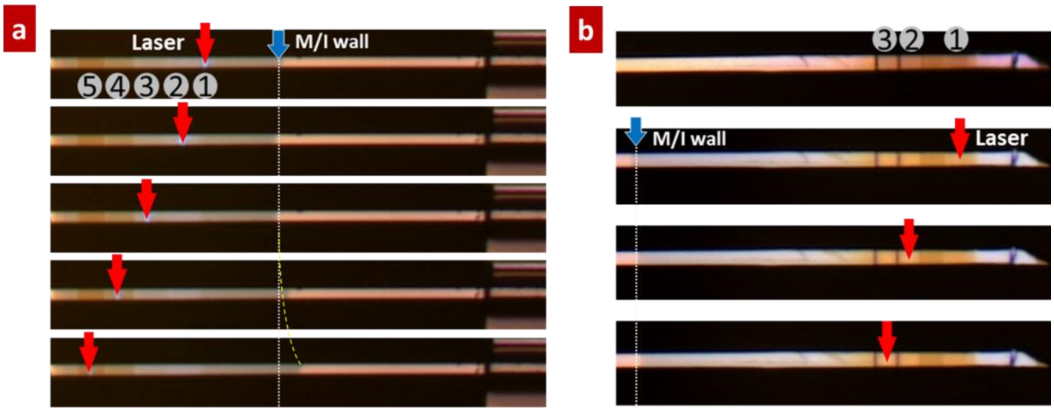

FIG.S1. Effects of Ga ion implantation on the optical absorbance. (a) The flat surface of a VO_2 microwire was milled to a depth of 200 nm with the constant operation current of 10 pA and different operation voltages of ②5kV, ③10kV, ④15kV and ⑤30 kV. The M/I wall is fixed at a certain position when the laser with a constant power focuses on the sections ①un-milled or milled with the voltage less than 15kV. Beyond 15kV, the M/I wall moves towards the root of a VO_2 microwire while the operation voltage is increased. The milled sections with the operation voltage larger than 15kV show a clear darker contrast compared with the smooth part in optical reflection; this indicates effectively enhanced light absorption. (b) The flat surface of a VO_2 microwire was milled to the depths of ①10 nm, ②100 nm, ③200 nm with a constant FIB milling condition (10 pA and 30 kV). The M/I wall is fixed at a certain position when the laser focuses on the milled sections with the constant power. The milled sections①-③show almost the same contrast as the smooth part in optical reflection, indicating the same light absorption.

3. Temperature dependent resistance of as grown VO_2 beam

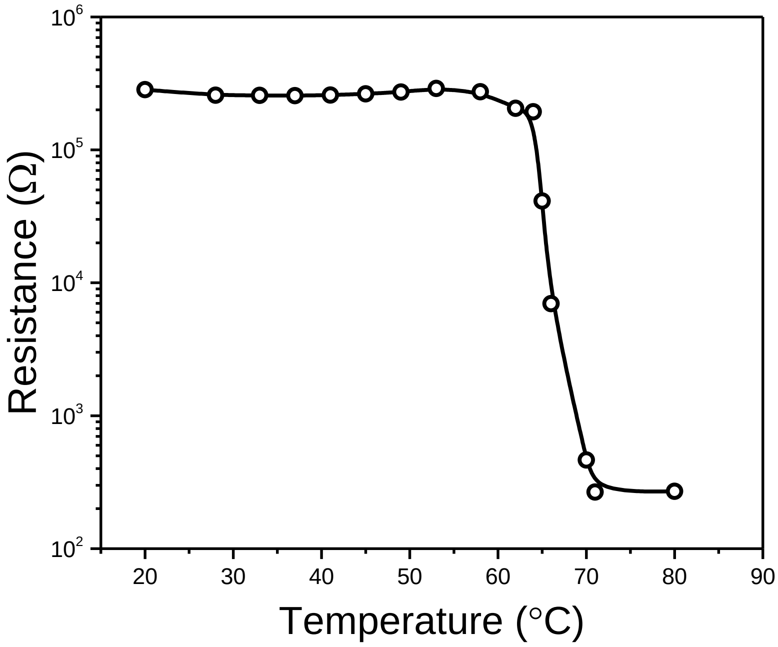

FIG.S2. Temperature-dependence (heating) of the resistance of un-doped VO_2 nanowire

The resistance of as-grown VO_2 nanowire indicates that the phase transition occurs at around 68 °C. The transition region is narrow which also proves the high quality of as-grown samples.

References

1. C. Cheng, K. Liu, B. Liu, J. Suh and J. Wu "Ultra-long, free-standing, single-crystalline vanadium dioxide micro/nanowires grown by simple thermal evaporation" *Appl. Phys. Lett.* 100 (2012) 103111

2. C. Cheng, D. Fu, K. Liu, H. Guo, S. Xu, S. Ryu, O. Ho, J. Zhou, W. Fan, W. Bao, M. Salmeron, N. Wang, C. P. Grigoropoulos, and J. Wu, Directly metering light absorption and heat transfer in single nanowires using metal-insulator transition, *Adv. Opt. Mater.*, 3（2015）336
